# Characteristics of Thermal Parameters and Some Physical Properties of Mineral Eutectic Type: Petalite–Alkali Feldspars

**DOI:** 10.3390/ma14237321

**Published:** 2021-11-30

**Authors:** Agata Stempkowska

**Affiliations:** Department of Environmental Engineering, Faculty of Civil Engineering and Resource Management, AGH University of Science and Technology, Mickiewicza 30 Av., 30-059 Kraków, Poland; stemp@agh.edu.pl

**Keywords:** high-temperature viscosity, melting temperature, mineral eutectic, petalite, sintering intervals

## Abstract

The aim of the research was to check whether the system of three fluxes based on lithium aluminium silicate and alkali feldspars has a eutectic point, i.e., with the lowest melting temperature. Lithium was introduced into the mixtures in the form of petalite, which occurs naturally in nature (Bikita Zimbabwe deposit). Using naturally occurring raw materials such as petalite, sodium feldspar, and potassium feldspar, an attempt was made to obtain eutectics with the lowest melting point to facilitate thermal processing of the mineral materials. In addition, the high-temperature viscosity of the mineral alloys and physical parameters such as density, linear shrinkage, and open porosity were studied. The study showed that in these systems, there is one three-component eutectic at 1345 °C, with the lowest viscosity of 1·10^5^ Pas and the highest density of 2.34g/cm^3^, with a weight content of petalite 20%, sodium feldspar 20%, and potassium feldspar 20%**.**

## 1. Introduction

**Figure 1:**
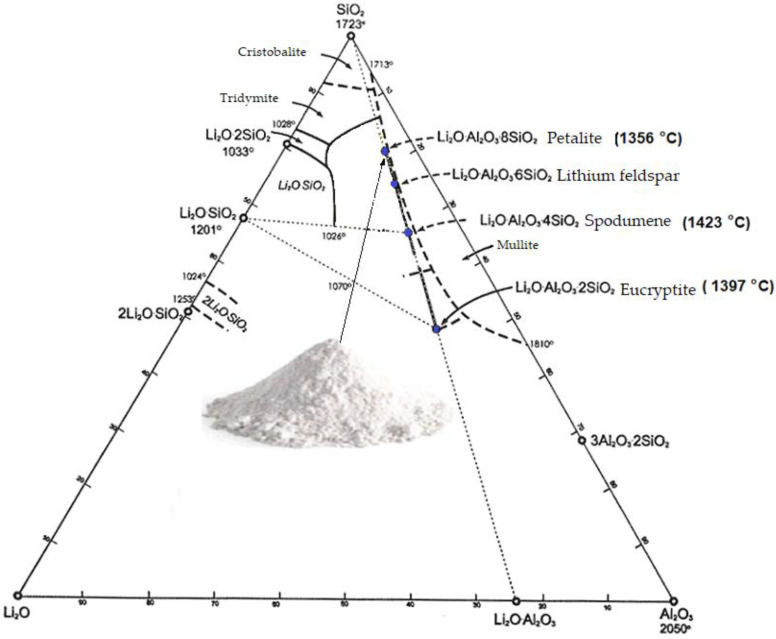
Diagram of lithium aluminium silicates.

Lithium is widespread and present in nature under various forms in about 145 minerals. However, the raw materials used industrially include only four of them: spodumene, lepidolite, petalite, and amblygonite. These are lithium silicates and phosphates, which are formed from transformations of pegmatites, and often, they co-occur with each other [1,2]. Lithium carbonate, obtained from lithium brines or from other lithium minerals, is also sometimes used. However, lithium in carbonate form, in high-temperature processes (sintering), can cause a problem with degassing of the resulting CO_2_ [3,4,5]. This article presents the characteristics of petalite, a natural variety of lithium aluminium silicate that does not generate CO_2_ emissions. Petalite is one of the varieties of lithium aluminium silicate with the general formula LiAl[Si_2_O_10_]. Its name comes from the Greek petalion meaning leaf or blade. The chemical composition in terms of oxides of the pure mineral is as follows: LiO_2_—4.86%, Al_2_O_3_—16.65%, and SiO_2_—78.48%. Often, trace amounts of magnesium, sodium, iron, or calcium are present in its composition. Of the lithium aluminium silicates shown in the binary diagram (Figure 1), only eucryptite and spodumene are well-studied compounds [1]. Petalite is sometimes called lithium orthoclase and is most likely solid solutions of the system: spodumene or SiO_2_. This is due to the small size of the Li^+^ cation compared to the Al^3+^ and Si^4+^ cations. This gives the possibility for the lithium ion to locate in both octahedral and tetrahedral gaps [6]. Nevertheless, in the present work, the influence of natural lithium aluminous silicate as a component of petalite mineral fluxes was determined.

**Figure 2:**
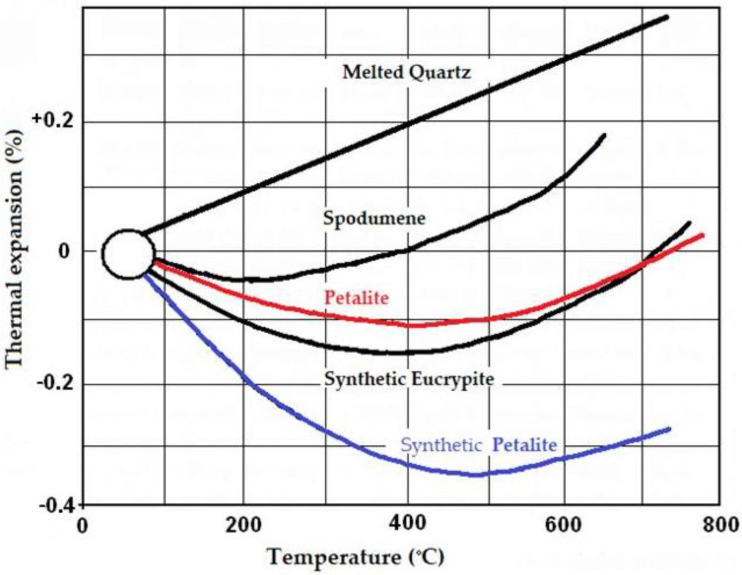
Changes in the coefficient of thermal expansion as a function of temperature of natural and synthetic varieties of lithium aluminium silicates relative to molten quartz.

Petalite crystallises in a monoclinic crystallographic system with the space group P2/a. It rarely forms tabular or bar-shaped crystals; much more often, it forms close-cropped crystals. It usually occurs in compact aggregates. It is a brittle mineral that is characterised by excellent flaking and forming a shell break. Its density ranges from 2.39 to 2.46 g/cm^3^, and its hardness is about 6.5 on the Mohs scale. Depending on the area of occurrence and the presence of admixtures, this mineral can be colourless or yellow, grey or white. However, it is usually transparent, and by grinding on its surface, one can see a multicoloured streak resembling a cat’s eye. At present, petalite, due to the presence of Al_2_O_3_ that increases scratch resistance, is most often used to produce special glasses such as cell phone screens [7,8]. Both petalite and spodumene are silicate minerals because they form the main structure. They are a valuable source of water-insoluble lithium. Petalite has the highest Li_2_O/Al_2_O_3_ ratio among natural minerals. It can also be a valuable flux additive because it increases their resistance to heat shock. Petalite itself has a near-zero coefficient of thermal expansion at about 700 °C and is a valuable component of mineral alloys because of its thermal properties [9,10]. As mentioned above, the most characteristic property of lithium aluminosilicates is the small thermal expansion coefficient of especially β-forms. As can be seen in Figure 2, the value of the thermal expansion coefficient ranges from a very small value for β-spodumene to even a negative value for the synthetic forms of synthetic β-eucryptite and petalite. The small value of the coefficient of thermal expansion is used to create materials that are extremely resistant to rapid temperature changes. Among other things, the negative coefficient of β-eucryptite of –7 × 10^−6^/°C is used in materials for special applications as compensation for the high coefficient of expansion of other components [11,12].

Alkali feldspars are typical fluxes with industrial applications. They are raw materials that are rich in alkali (K_2_O + Na_2_O), which are mainly bound in them as aluminosilicates. The sodium substrate, albite, usually contains calcium impurities. This is because plagioclase forms the albite–anorthite series. An exception is the raw material under the name Albitte 5 (Turkey), which contains less than 1% calcium oxide and can be treated as a pure mineral [13,14]. This raw material has an average density of 2.62 g/cm^3^ and has a melting point of 1120–1200 °C, it also has the lowest high temperature viscosity compared to other feldspars. Since sodium feldspar melts congruently, it causes more deformation of the material but is considered to be a better flux than potassium feldspar due to its lower melting point. The potassium carrier is orthoclase, which has an average density of 2.55 g/cm^3^ and a melting point of about 1170 °C. During sintering, the molten silicates interact with the solid phase and partially dissolve it. This phenomenon begins at a temperature of about 1150 °C. It fills the air voids of the mineral material and, consequently, its compaction causes the silicate to melt. In technological processes, the most favourable ratio of K_2_O to Na_2_O is 2. In melt systems, systems with Na_2_O to K_2_O predominance are usually preferred, which is justified by the congruent melting of sodium feldspar [6,15,16,17]. The melting points of alkali feldspars are significantly lower than petalite; however, the contribution of lithium aluminium silicate, in a synergistic effect, can further lower the melting point [18,19,20]. Fluxes introduce alkali metal oxides into the mineral mass, and all these fluxes melt at temperatures above 1100 °C and affect the evolution of the crystalline phases and consequently the properties of the final materials. Lithium silicates partially replace feldspars. In the industry, they are used for porcelain (bone china) sanitary or technical ceramics [21,22,23]. In general, lithium is associated with applications in green energy storage technologies and is becoming a crucial metal of importance [24,25]. However, there is little research on the use of lithium-containing raw materials as aggressive fluxes, and this is an important topic that is recognized relatively little.

The main objective of this work was to determine the image of phase transformations of a three-component system, containing alkalis such as Na, K, and Li. The presence of Li_2_O reacting with other oxides and silicates created a liquid phase with lower viscosity, which favours densification by sintering with viscous flow and increased mechanical strength. Large firing intervals were achieved with no deformation upon firing, which is not an easy task.

## 2. Materials and Methods

### 2.1. Layout of Flux Raw Materials

**Figure 3:**
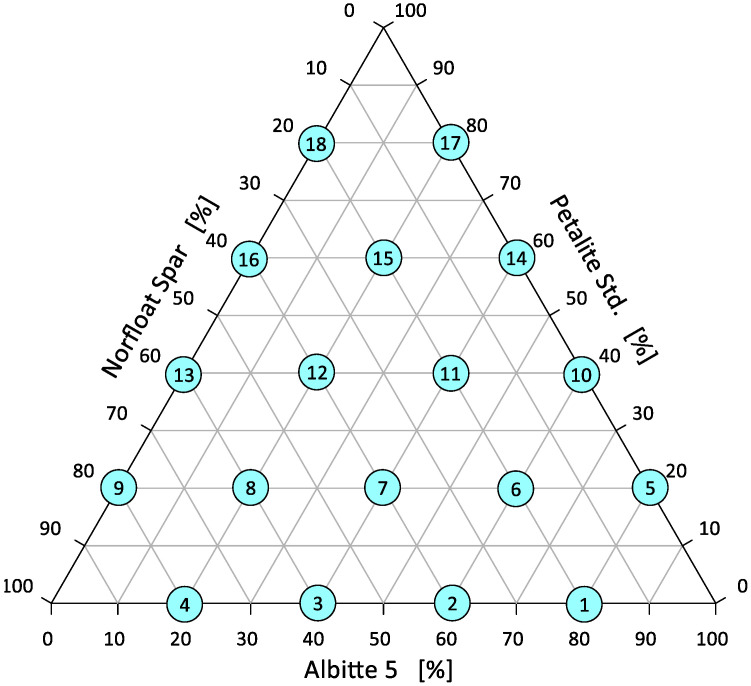
Distribution of samples (depending on weight composition) in the Petalite–Na–feldspar–K–feldspar triangle.

Commercially available natural raw materials containing alkaline elements were used in this study. For lithium aluminium silicate, commercial Petalite Std was supplied by Otavi Minerals, Neuss, Germany. For feldspars, Albitte 5 and Norfloat Spar were supplied by Otavi Minerals, Germany. The compositions of the individual raw materials in terms of oxides are shown in Table 1. Basic sets were prepared differing by 20% of the individual components as shown in Figure 3. Regardless of the degree of grinding of the individual components, all sets were additionally homogenised for about 15 min in a mill (Fritsch Pulvrisette, Idar-Oberstein, Germany). The average grain distribution of the sets did not exceed 50 µm.

### 2.2. High-Temperature Microscopy

**Figure 4:**
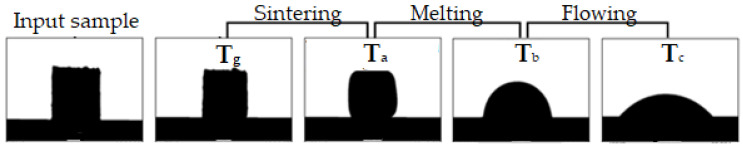
Method for determining characteristic temperatures and intervals in a high-temperature microscope, using sample No. 1 as an example.

A high-temperature microscope EM301 (Hesse-Instruments, Osterode am Harz, Germany) was used to measure the characteristic temperatures. The measurement conditions were as follows: temperature increments of 80 °C/min to 650 °C and 10 °C/min in the temperature range from 650 to 1500 °C. As a result of the permanent observation of the sample during which it changes its dimensions, characteristic temperatures can be determined (Figure 4), [26,27]. The first change is referred to as the sintering onset temperature T_g_. Then, when the corners of the specimen become rounded, the softening temperature T_a_ is determined. This stage is considered as the completion of sintering. The formation of mineral eutectic occurs at the temperature of hemisphere formation T_b_. In other words, it is the melting point. When the base of the sample reaches 200% of the original size or the height is reduced to 1/3, the spreading temperature T_c_ is determined.

The differences between the individual characteristic temperatures allow the determination of sintering, melting, and flowing intervals. Sequentially, the sintering interval is defined as the difference between the corner rounding temperature T_a_ and shrinkage temperature T_g_. Then, the melting interval is the difference between the hemisphere temperature T_b_ and corner rounding temperature T_a_. Finally, the flowing interval is the difference between spreading temperature T_c_ and hemisphere temperature T_b_.

The temperature microscope allows the determination of a number of thermal properties of materials. These include not only the characteristic temperatures written above but also decomposition temperatures, sublimation temperatures, phase transition temperatures, etc. [26]. The observation of changes in the dimensions of the specimen is continuous as a function of temperature, and therefore, the melt viscosities or wettability relative to the surface can also be determined [28]. The graphical visualisation of the data obtained from the high-temperature microscope seems interesting. Surfer 19 software from GoldenSoftware was used for this purpose. The test results presented are the averages of three measurements.

### 2.3. Physical Parameters of the Melt

Viscosity is the resistance force that inhibits the movement of fluid components relative to each other. The Vogel–Fulcher–Tamman (VFT) equation in logarithmic form (1) allows the determination of the dependence of viscosity on melt temperature. One of the simplest viscosity models was chosen because the measurement and determination of this parameter is very difficult due to the variable chemical composition of raw materials [29,30,31,32,33]

(1) $$\log\eta =A+\frac{B}{T-T_{0}}$$

where

η—viscosity (Pa·s);

A, B—constants depending on the chemical composition of the set;

T—temperature (K);

T_0_—temperature VFT, sintering temperature (K).

Considering that petalite, spodumene, and other lithium silicates can form solid solutions with each other, it is interesting to study the change in sinter density, open porosity, and linear shrinkage as a function of temperature. These measurements were made using 30 mm diameter samples formed by pressing. The pellets were fired in a gradient furnace LSP30/13, from LAC, Brno, Czech Republic.

The apparent density is the ratio, expressed as a percentage, of the mass of the porous material to the volume occupied by it. Density was determined by hydrostatic weighing using Formula (2):

(2) $$d_{p}=\frac{m_{s}}{m_{s}-m_{w}}\cdot d_{c}$$

where

d_p_—apparent density (g/cm^3^);

m_s_—dry sample mass (g);

m_n_—mass of sample saturated with water (after boiling) (g);

m_w_—weight of sample saturated with water (g);

d_c_—density of water at the measurement temperature (g/cm^3^).

Linear shrinkage was determined from the changes in diameter of pastilles fired at different temperatures. Measurements were made by measuring the diameter three times in different directions and using Formula (3):

(3) $$S_{w}=\frac{M-S}{M}\cdot 100$$

where

S_w_—linear shrinkage (%);

M—diameter of the pastille after forming and drying (mm);

S—diameter of the pastille after firing (mm).

Open porosity is the ratio, expressed as a percentage, of the volume of pores filled with water during the test to the volume of the sample. Porosity was calculated from Formula (4):

(4) $$P_{0}=\frac{m_{n}-m_{s}}{m_{n}-m_{w}}\cdot 100$$

where

P_o_—open porosity (%);

m_s_—dry sample mass (g);

m_n_—mass of the sample saturated with water (after boiling) (g);

m_w_—weight of the sample saturated with water (g).

## 3. Results and Discussion

### 3.1. Characteristic Temperatures and Their Intervals

**Figure 5:**
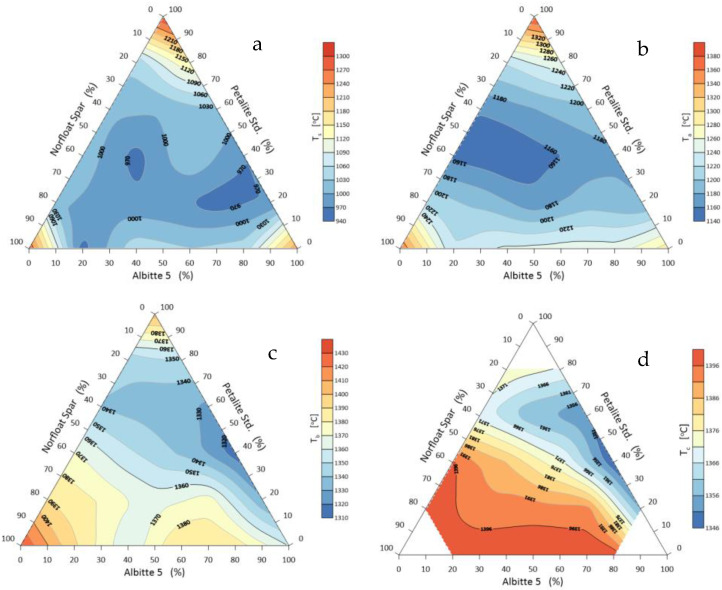
Sintering maps of the flux system: Petalite Std/Albitte 5/Norfloat Spar, as a function of temperature: (**a**) onset of shrinkage, (**b**) corner rounding, (**c**) hemisphere and (**d**) spread.

The beginning of shrinkage of each set during heating is the onset of sintering, and the range until rounding of the corners is referred to as the sintering interval. Two mechanisms cause the volume change in samples. The first, occurring at about 1000 °C, is related to thermal expansion of the grains, which induces about 1.5% volume swelling of the samples. In the next heating step, contraction occurs due to sintering, melting, and elimination of the gas phase [34,35]. The characteristic temperatures of the tested sets are shown in Figure 5.

**Figure 6:**
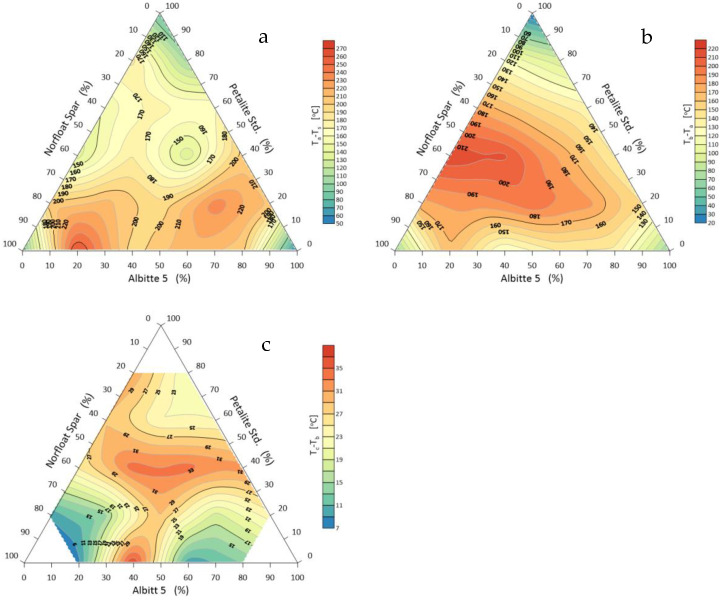
Intervals of (**a**) sintering, (**b**) melting, and (**c**) spreading of the Petalite Std/Albitte 5/Norfloat Spar flux system.

It appears that during the initial heating period, the beginning of shrinkage is greatest when all components of the flux system are equal. This shrinkage starts at the temperature level of 900 °C. In a further stage, that is, at the start of the rounding of the corners, a eutectic is formed with a composition of, respectively, 20% Albitte 5, 20% Petalite Std, and 60% Norfloat Spar (set 6) (Figure 3). The ternary eutectic of onset of flow is at 1346 °C. The lowest temperature of hemisphere formation occurs with an increased proportion of sodium feldspar >80% and equal proportions of petalite and potassium feldspar. At this point, the melting temperature of the system is about 1320 °C. The eutectic point shifts towards higher petalite contents, while the effect of sodium feldspar on the melting point of the system is highlighted. This effect can be clearly observed by analysing the sintering, melting, and flow time intervals (Figure 6). The softening stage is usually defined as the difference between the shrinkage onset temperature and the hemisphere temperature. This stage causes a large shrinkage which in the case of mineral materials leads to deformation of the products, but for the alloy, there is no such danger. This is justified by the usually narrow transition interval between the solid and liquid states.

As for the sintering interval of the system, which is calculated from the shrinkage starting temperature to the corner rounding temperature (Figure 4), it is technologically less relevant for fluxes. It is important in the sintering processes without the participation of the liquid phase. However, the smaller this interval is, the faster the system goes to the melting stage. The study shows that the sintering interval is smaller the closer the composition of the sets approaches the eutectic of melting, which is the centre of the composition triangle. Two increased ranges of sintering interval can also be observed with a small contribution of petalite. The wide melting interval of the studied system occurs when there is no sodium feldspar. This fact highlights the significant influence of Albitte 5 and the smaller Petalite Std on the melting process, although in the case of melt flow, the smallest interval was obtained with an increased proportion of Norfloat Spar >70%. This fact is difficult to explain on the basis of previous studies.

### 3.2. High-Temperature Viscosity, Density, Open Porosity, and Linear Shrinkage

Viscosity flow occurs at the beginning of sintering involving the liquid phase and during the sintering of bodies. This process involves a group movement of atoms, and the viscosity coefficient η, as a material constant, determines the rate of this process [36,37]. The viscosity coefficients, based on Equation (1), correspond to the following temperatures: softening (rounding of corners) and melting (hemisphere) (Figure 4). Meltability is determined on the basis of changes in shape or dimensions of the sample observed during the measurement. The compositions selected for measurement in the current study will allow a systematic investigation of the effects of different components on melt viscosity. The range of measurements was chosen taking into account limiting factors such as the liquidity temperatures of the different mineral compositions and the maximum operating temperature of the furnace. The results are presented in Table 2.

**Figure 7:**
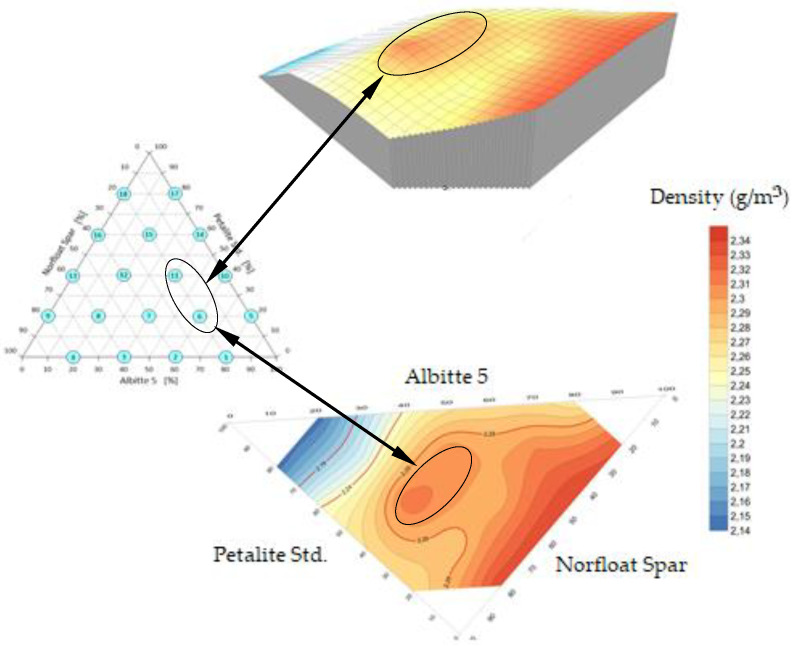
Spatial visualisation of density changes in Petalite Std/Albitte 5/Norfloat Spar system.

Viscosity is one of the most significant physical properties of mineral alloys that must be controlled to improve the efficiency of the sintering processes. The effect of temperature and composition on the viscosity of silicate melts is very important. From the viscosity coefficient measurements, it can be seen that the addition of petalite has a clear effect on reducing the viscosity of the alloys. The lowest viscosity was obtained for sets 6 and 11 containing 20% and 40% Petalite Std, respectively. Similarly, sets 6 and 11 have the lowest viscosity attainment temperature of 106 Pa·s. In the high-temperature range (η < 106 Pa·s), the viscosity reduction is directly related to the weakening of the spatial network of silicates [26]. The recrystallisation process already occurring when the material is heated to the sintering temperature in principle no longer has a direct effect on the density changes of the sinter. Pore disappearance and grain growth during the sintering of porous materials are parallel and interacting processes. Sintering creates permanent bonds between particles. This bonding reduces the surface energy by reducing the free surface area. In this process, grain boundaries are partially eliminated by grain growth and pore volume is reduced, resulting in a condensed mass [38,39]. In this way, an increased density of the alloy is obtained as well as an improvement in its strength [40,41]. Figure 7 shows a visualisation of the density variation of the flux system. The highest density was obtained in the sets without Petalite and in the middle of the system roughly corresponding to sets 6 and 11 with Petlite Std content composition of 20% and 40%, respectively. Densification occurs because a reaction between the components of the system is initiated. In the final sintering step, densification occurs by removing closed pores due to grain growth mainly in the presence of a small amount of liquid phase, where the low melt viscosity is crucial (Table 2).

**Figure 8:**
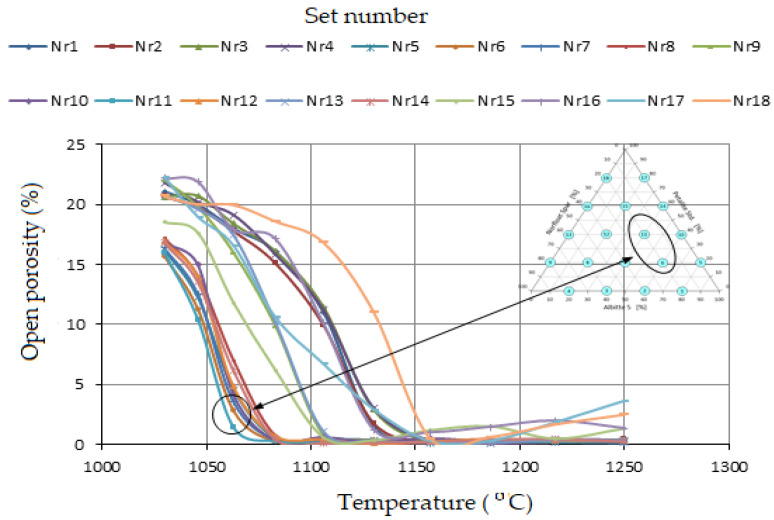
Change in open porosity of samples as a function of temperature.

Density has a direct relationship to open porosity. During the sintering process, samples undergo the maximum possible consolidation. The mechanism of the process includes the regrouping of mineral grains, the growth of crystallites, and the reduction of the proportion of pores. The area of contact angles between grains increases due to mass transfer into voids [42]. The maximum packing at temperatures around 1070 °C was obtained for sets 6 and 11, which had an open porosity 1.5 and 2.5%, respectively (Figure 8). Above a temperature of 1150 °C, swelling was observed in some sets. These were mainly sets 17 and 18 (Figure 8) containing 80% petalite.

**Figure 9:**
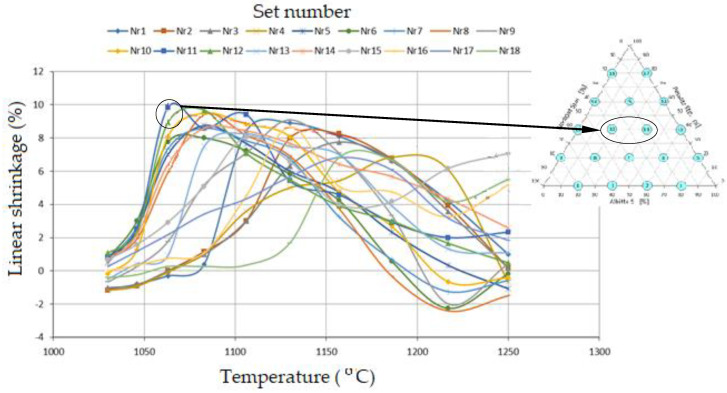
Change of linear shrinkage of sets as a function of temperature.

The volume changes are most likely due to polymorphic transformations of lithium silicates of a series of petalite–spodumene–SiO_2_ solid solutions (Figure 1). The volume of β-spodumene is 17% larger than that of the γ variety and as much as 26% larger than that of the α variety [43]. The graphs of linear shrinkage changes of sinter are shown in Figure 9. The highest linear shrinkage of 9–10% was observed in sets 11 and 12. Unfortunately, the samples are deformed during gradient sintering, which makes the measurement difficult.

The use of Petalite Std has a range of application advantages. The replacement of some Na and K feldspars with lithium silicate lowers the melting temperature and increases the temperature range of the process. A low-viscosity liquid phase is also obtained. Lowering the sintering temperature means significant savings in fuel consumption. In addition, a higher sinter density is achieved. Possible complications are deformations of the shapes due to polymorphic changes of silicates at high temperatures.

## 4. Conclusions

- The first onset of shrinkage T_s_ (the beginning of sintering process) was observed for set 6 at about 900 °C.
- The eutectic melting point of the three-component mineral system; petalite (Petalite Std)–potassium feldspar (Norfloat Spar)–sodium feldspar (Albitte 5) occurred at 1346 °C at 20/60/20% weight proportions, respectively.
- The eutectic region occurs for sets 6 and 11 and 15 for petalite contents of 20%, 40%, and 60% by weight and a fixed sodium feldspar content of 20 wt %.
- An increase was observed in the density of samples in the centre of the triangular compositions in sets 6 and 11, with petalite contents of 20 wt % and 40 wt %, respectively.
- The lowest viscosity values of 1 × 10^5^ and 1.26 × 10^5^ were similarly observed for samples 6 and 11.
- In further works, the author will present the influence of Ca- and Mg-containing raw materials on the Li-Na-K flux system.

## Figures and Tables

**Table 1:** Chemical composition of applied raw materials.

Chemical Composition	Norfloat Spar	Albitte 5	Petalite Std
		wt %	
SiO_2_	65.9	67.1	77.08
Al_2_O_3_	18.5	18.8	16.56
CaO	0.5	0.6	0.14
MgO	0.1	1.3	0.04
TiO_2_	<0.05	0.36	0.01
Fe_2_O_3_	0.23	0.7	0.05
MnO	<0.05	<0.05	0.01
P_2_O_5_	0.06	0.13	0.01
**Na_2_O**	**2.9**	**9.5**	**0.62**
**K_2_O**	**12.0**	**0.2**	**0.42**
**Li_2_O**	**-**	**-**	**4.28**
LOI	1.02	1.49	0.76

**Table 2:** High-temperature viscosity and temperatures at a specific viscosity level of the Petalite Std/Norfloat Spar and Albitte 5 system.

Set Number	High-Temperature Viscosity Pa∙s			Temperature at Specific Viscosity Level °C		
	1400 °C	1500 °C	1600 °C	η·10^10^	η·10^8^	η·10^6^
1	1.26 × 10^14^	5.01 × 10^6^	3.98 × 10^5^	1446	1459	1540
2	5.01 × 10^10^	3.98 × 10^6^	6.03 × 10^5^	1415	1445	1550
3	1.26 × 10^8^	3.63 × 10^6^	5.89 × 10^5^	1359	1407	1555
4	6.31 × 10^7^	3.16 × 10^6^	5.62 × 10^5^	1350	1391	1556
5	1.29 × 10^7^	1.26 × 10^6^	7.41 × 10^5^	1273	1340	1502
6	**1.58 × 10^6^**	**1.58 × 10^5^**	**1.00 × 10^5^**	1317	1339	**1425**
7	2.00 × 10^7^	1.41 × 10^6^	7.08 × 10^5^	1320	1375	1525
8	7.94 × 10^6^	1.58 × 10^6^	6.31 × 10^5^	1250	1336	1510
9	5.01 × 10^6^	4.47 × 10^5^	1.78 × 10^5^	1310	1349	1460
10	1.26 × 10^7^	1.12 × 10^6^	2.34 × 10^5^	1300	1352	1499
11	**7.94 × 10^6^**	**5.62 × 10^5^**	**1.26 × 10^5^**	1350	1370	**1450**
12	7.08 × 10^6^	1.00 × 10^6^	2.24 × 10^5^	1247	1320	1475
13	6.31 × 10^6^	7.08 × 10^5^	2.24 × 10^5^	1269	1325	1461
14	1.58 × 10^7^	8.91 × 10^5^	1.99 × 10^5^	1352	1360	1480
15	6.31 × 10^6^	7.94 × 10^5^	1.82 × 10^5^	1275	1339	1491
16	2.00 × 10^7^	1.12 × 10^6^	2.45 × 10^5^	1336	1358	1502
17	3.98 × 10^6^	1.26 × 10^6^	7.90 × 10^5^	1213	1250	1570
18	4.79 × 10^6^	1.12 × 10^6^	3.98 × 10^5^	1299	1350	1512

## Data Availability

The data presented in this study are available on request from the author.
